# Asbestos and Other Hazardous Fibrous Minerals: Potential Exposure Pathways and Associated Health Risks

**DOI:** 10.3390/ijerph19074031

**Published:** 2022-03-29

**Authors:** Terri-Ann Berry, Elena Belluso, Ruggero Vigliaturo, Reto Gieré, Edward A. Emmett, Joseph R. Testa, Gregor Steinhorn, Shannon L. Wallis

**Affiliations:** 1 Environmental Solutions Research Centre, Unitec Institute of Technology, Auckland 1025, New Zealand; tberry@unitec.ac.nz (T.-A.B.); gsteinhorn@unitec.ac.nz (G.S.); 2 Department of Earth Sciences and Interdepartmental Centre for Studies on Asbestos and Other Toxic Particulates, University of Torino, 10124 Turin, Italy; elena.belluso@unito.it (E.B.); ruggero.vigliaturo@gmail.com (R.V.); 3 Earth and Environmental Science, University of Pennsylvania, Philadelphia, PA 19104, USA; giere@sas.upenn.edu; 4 Perelman School of Medicine, University of Pennsylvania, Philadelphia, PA 19104, USA; emmetted@mail.med.upenn.edu; 5 Cancer Signaling and Epigenetics Program, Fox Chase Cancer Center, Philadelphia, PA 19111, USA; joseph.testa@fccc.edu

**Keywords:** asbestos fibres, erionite, malignant mesothelioma, exposure, asbestiform

## Abstract

There are six elongate mineral particles (EMPs) corresponding to specific dimensional and morphological criteria, known as asbestos. Responsible for health issues including asbestosis, and malignant mesothelioma, asbestos has been well researched. Despite this, significant exposure continues to occur throughout the world, potentially affecting 125 million people in the workplace and causing thousands of deaths annually from exposure in homes. However, there are other EMPS, such as fibrous/asbestiform erionite, that are classified as carcinogens and have been linked to cancers in areas where it has been incorporated into local building materials or released into the environment through earthmoving activities. Erionite is a more potent carcinogen than asbestos but as it is seldom used for commercial purposes, exposure pathways have been less well studied. Despite the apparent similarities between asbestos and fibrous erionite, their health risks and exposure pathways are quite different. This article examines the hazards presented by EMPs with a particular focus on fibrous erionite. It includes a discussion of the global locations of erionite and similar hazardous minerals, a comparison of the multiple exposure pathways for asbestos and fibrous erionite, a brief discussion of the confusing nomenclature associated with EMPs, and considerations of increasing global mesothelioma cases.

## 1. Introduction

Although many articles have been published on the topic of asbestos-related diseases (ARD) (nearly 15,000 from 1991 to 2016), a downturn in the topic’s popularity has been highlighted recently, with concerns about the declining emphasis on public areas in ARD-related literature [1]. The global burden of disease (GBD) estimates that occupational and environmental exposure to asbestos may have been significantly underestimated [2], and in the coming decades, the incidence rates of ARD are expected to peak [1]. This research has included consideration of exposure risks from other elongated mineral particles (EMPs) [1,2]. It is therefore important, from a public health perspective, that we continue to investigate other exposure pathways and possible causes of ARDs such as malignant mesothelioma (MM), asbestosis, pleural abnormalities and bronchogenic carcinomas, previously attributed to asbestos [3]. The dimensional characteristics of asbestos fibres are important physical parameters linked to respiratory disease, and this has led to studies of other EMPs of similar dimension and habit [4], and often with comparable chemistry, and/or surface characteristics. For example, fibrous fluoro-edenite has been found to be responsible for excess MM in Biancavilla, Italy [5], and fibrous erionite, a fibrous mineral that belongs to a group of minerals called zeolites, has been assessed by the International Agency for Research on Cancer (IARC) as a Group-1 human carcinogen [6].

Although the exact mechanisms for the carcinogenic response caused by inhaled asbestos particles and other elongate mineral particles (e.g., fibrous fluoro-edenite and fibrous erionite) are not fully understood, the available evidence supports that harm may be caused by both long-term and short-term exposure [7,8]. EMPs include asbestiform (non-asbestos classified minerals which are similar to asbestos in terms of morphology and properties [4,9]) and non-asbestiform minerals as well as cleavage fragments of non-asbestiform variants of asbestos minerals [10].

For the purpose of this article, the term “EMP” will be a substitute for the term “fibre” [4] and refers to a mineral particle that exhibits an aspect ratio (L/w) of ≥3:1, with a length (L) > 5 µm. The replacement of the term “fibre” with the term “EMP” was made specifically to include both the asbestiform and the non-asbestiform habits, which meet the dimensional criteria specified by NIOSH (2011) [4]. The term “fibre” will be used as defined in Belluso et al. (2017) [9], i.e., elongated particles with uniform parallel sides and geometrical faces exhibiting L/w ≥ 3:1, L ≥ 5 µm, and w ≤ 3 µm. This is intended only as a mineralogical definition of a habit, and not as the term identifying a “regulated fibre” with specific dimensional parameters. The term “asbestos particles” will be used when referring to chrysotile asbestos, riebeckite asbestos (crocidolite), grunerite asbestos (amosite), tremolite asbestos, anthophyllite asbestos, and actinolite asbestos [9]. The term asbestiform (a subset of fibrous) will be used to identify EMPs with the same dimensional criteria as described for “fibre” and at least one of the following characteristics: large EMP length, small EMP thickness, separability, flexibility, and a parallel arrangement of the EMP observed in an unprocessed sample [11].

Asbestos is banned in many countries around the world, including Japan, Australia and all countries in the European Union. However, chrysotile continues to be mined and used worldwide, especially in Asia and Russia, with the top producers being Russia, Kazakhstan, China and Brazil [12], and the leading importers being India, China and Indonesia [13]. There is also increasing evidence of environmental exposure to asbestos from both geological and anthropogenic sources [14,15]. Previous research on asbestos has yielded valuable information on how these EMPs cause cancer and fibrosis [3,16,17,18,19]. This foundation has been built upon to assist research on other EMPs which share some of the characteristics linked to malignancy. For example, fibrous erionite is an EMP that belongs to a group of silicates called zeolites. This mineral may crystallize as prismatic particles, nm-µm in width and µm-mm in length, and the disturbance of rocks containing this mineral can generate airborne particles similar in size and shape to those of asbestos. Nearly 40 years ago, erionite in its fibrous form was shown to have genotoxic activity [20], and subsequent studies demonstrated that fibrous erionite cause tumours in rats at much higher rates than any amphibole or serpentine asbestos fibres tested [21,22].

For many EMPs, a lack of pure mineral handling has resulted in health risks that are perceived to be far lower than those from exposure to asbestos-contaminated materials and soils. Alternatively, these health risks have simply not been considered at all. However, exposure to harmful EMPs such as fibrous erionite and fibrous offretite (the latter is another member of the zeolite group, very similar to the first and distinguishable only by in-depth analyses [23]) may present us with new risk groups for diseases such as fibrosis, MM and lung cancers [21,24,25]. Around 40 years ago, estimates of the comparative risk of adverse health effects that might result from exposures to various EMPs were made by the National Research Council (NRC), Atlanta, GA, USA [26]. Although this work considered many different types of EMP, it was concluded that the biggest risk was associated with exposure to chrysotile, based mainly on the greater opportunities for exposure to airborne particles of respirable size [26]. Fibrous erionite of respirable size was discovered in deposits in the western United States, but measurements of local air did not yield significant fibrous erionite concentrations despite the potential link to mining and natural weathering in the area. It was also concluded that the population exposed would be small, but this may be because the study locations, i.e., Rome, Oregon and East Gate, Nevada (previously examined by Wright et al., 1983 [27]) were two small unincorporated communities (settlements not governed by their own local municipal corporation), which are sparsely populated. The lack of fibrous erionite-related MM in the western area of the United States has been linked to this factor by other authors [28].

**Figure 1:**
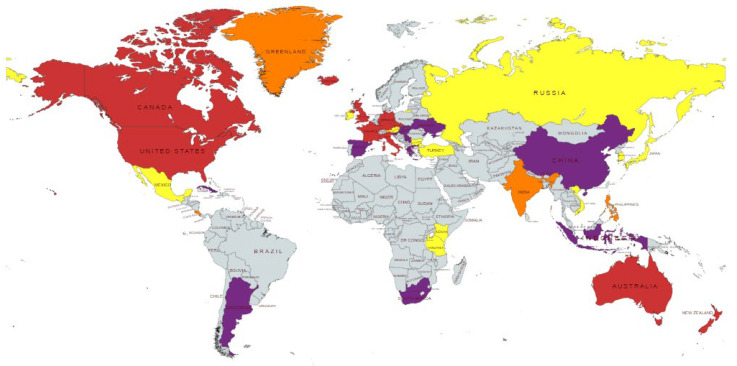
Most common identified locations of zeolites and specific occurrences of erionite and offretite (without distinction between habits). Key: purple = zeolites (general); yellow = erionite (only); orange = offretite (only); red = erionite and offretite. References: Zeolite Mining–[31,32,33]. Erionite deposits–[34,35,36,37,38,39,40,41,42,43,44] and mentioned in [45]. Offretite deposits–[36,46].

For erionite and offretite, limited geographical distribution does not appear to be the reason for the apparent absence of proven links between exposure and MM diagnosis. Since the 1960s, erionite has been identified on all seven continents and in more than 25 countries (Figure 1). Fibrous offretite can also cause adverse effects on human health and may be found within erionite clasts [29]. Despite the fact that fibrous erionite is more potent at causing MM than asbestos [17,22,30], exposure is less widespread, having not been mined and/or used to the same extent. Nevertheless, anthropogenic activities which could result in dispersal of erionite particles into highly populated areas may require tighter controls and mitigation methods to prevent the creation of future cancer epidemics. This study will explore known and potential human exposure pathways in urban environments to asbestos and erionite minerals. It will also explore global trends of MM cases and discuss why exposure to erionite has demonstrated high variability in terms of carcinogenic response.

## 2. Exposure Pathways

### 2.1. Asbestos

Globally, the majority of diagnosed MM cases over the last 50 years have been attributed to occupational exposure, however, there are a significant number of cases arising from other exposure pathways, including para-occupational, domestic and environmental exposure [8]. 

#### Exposure Pathways

Occupational exposure (asbestos industry), e.g., asbestos mining, asbestos containing material (ACM) production and manufacture;

Occupational exposure (non-asbestos industry), e.g., trades (plumbing, electrical, heating), automotive and ship-building industries, reclamation of ACM;

Domestic and para-occupational exposures (risks associated with living with those working in asbestos-related industries and home-based exposure), e.g., work clothes and home renovation;

Environmental exposure (both anthropogenic and natural environmental exposure), e.g., neighbourhood exposure to mining industry/manufacturing plants, asbestos substrates or outcrops of asbestos containing rocks (also named natural occurrence of asbestos-NOA), demolition, deterioration of buildings, and emergency scenarios including fires and earthquakes.

Over the past century, asbestos has been identified as an inhalation hazard in many occupational environments [47], including asbestos mining and manufacturing. Although the use and production of asbestos and asbestos-contaminated materials (ACM) have been banned in 67 countries [48], the risk of exposure still exists from ACM, which remains within industrial environments, public, private and school buildings, and homes. Within the non-asbestos industry, there are also many occupations that are considered to be high risk in terms of exposure which include shipbuilding, plumbing, carpentry and other trades [49] and reclamation works [50]. Rake et al. (2009) [49] used data obtained during interviews to estimate the risks and number of MM cases caused by specific occupational (non-asbestos industry) and environmental exposure in the United Kingdom (UK). This was the first population-based study and the largest worldwide, and the investigators concluded that UK carpenters suffered the highest risk followed by non-construction high-risk jobs (e.g., dock workers) [49]. An already dire prediction that 1 in 10 of all British carpenters born in the 1940s may die of cancer caused by asbestos was made worse by a study which showed that many tradespeople (plumbers in this instance), do not recognise the friable materials that they still sometimes encounter [51]. Material reclamation within an already high risk non-asbestos industry, namely shipbuilding, has been predicted to cause many deaths from mesothelioma in the future. India is responsible for close to 50% of worldwide ship recycling. A study by Singh et al. (2020) [52] estimated that nearly 15% of the total workforce engaged in ship recycling will suffer from mesothelioma, resulting in over 4500 mesothelioma deaths amongst workers from the period 1994 to 2002. However, the list of high-risk non-asbestos occupations is not-exhaustive as recent evidence highlights. A comprehensive review of published epidemiologic studies indicated that sailors are also at high risk of asbestos-related diseases and demonstrate elevated morbidity and mortality from mesothelioma and other ARDs [53].

**Figure 2:**
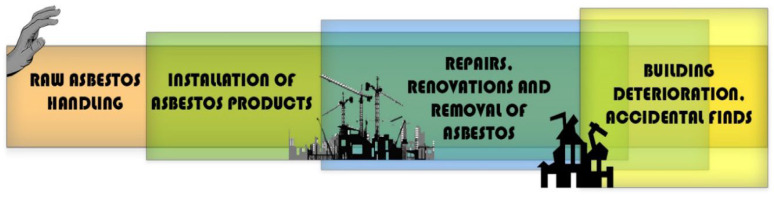
The four waves of asbestos exposure (figure based on Landrigan, 1991 [55]) and reproduced from Wallis et al., 2020 [54]).

The various epidemiological “waves” (Figure 2) of human exposure to asbestos have historically passed through raw asbestos handling, installation of products, repairs, renovations and removal of asbestos through to building deterioration, accidental finds and issues with long-term secure disposal [54].

Landrigan (1991) [55] raised concerns about the effects of short or long-term exposure to asbestos in the home or the workplace which was labelled as the third wave of exposure. Olsen et al. (2011) [56] reviewed all cases of diagnosed MM from 1960 to 2008, using the Western Australian Mesothelioma Registry. They concluded that asbestos exposure during home renovation is an increasing problem in Western Australia (WA), with associated MM cases appearing to show a shorter latency period, compared to exposure pathways. In 1981, the first case of MM associated with exposure attributed to home maintenance and renovation in WA was identified [56]. In this study, home renovators represented the largest proportion for all non-occupational cases. From 2005 to 2008, 8.4% of MM cases in men and 35.7% of MM cases in women were attributed to home renovation, and this has shown an upward trend over the last 10 years [56]. While many countries have produced codes of practice for the safe removal of asbestos, there is less specific information available for home renovations, individual tradesmen and other small operators [57,58].

Within the household contact pathway lies para-occupational or “take-home” exposure which has been recognised for the past 60 years [47]. In response to the lack of quantitative data available to characterise the para-occupational risk to asbestos, Sahmel et al. (2014) [47] examined airborne chrysotile concentrations produced during the handling of contaminated work clothes. This study used simulated occupancy with mannequins and a combination of Phase Contrast Microscopy (PCM) and Transmission Electron Microscopy (TEM) analysis, which were used to calculate lifetime cumulative dose [47]. Although the cumulative chrysotile doses for clothes handling were below or consistent with those for ambient or background chrysotile (over a 70-year lifespan), Sahmel et al. (2014) [47] noted that other studies had pointed to an increased risk of disease. It was suggested that this may have been due to the inclusion of other fibre types (mainly amosite or mixed fibres) in previous research [47]. In contrast, the UK mesothelioma case study previously described by Rake et al. (2009) [49], found that the only significant non-occupational association occurred from living with a potentially exposed worker before 30 years of age.

Living or spending time in or near a building with ACM does not necessarily present a health risk from domestic exposure. A study of over 750 buildings in the USA using TEM analysis concluded that in-place ACM does not result in elevated airborne asbestos concentrations or a significantly increased risk to building occupants [59]. While this study did not assess variability in ACM degradation, it did compare outdoor/indoor samples from ACM-containing buildings that were not significantly different [59]. To a certain extent, this was further supported by a recent assessment of the asbestos exposure level and carcinogenic risk from corrugated asbestos-cement slate roofs in Korea [60]. This study reviewed Korean literature to estimate the concentration of airborne asbestos from ACM roofs. The excess lifetime cancer risk for the indoor exposure and occupational dismantling and demolition was estimated to be of medium risk; however, caution was issued, as there is no threshold for carcinogenicity related to asbestos [60]. Campopiano et al. (2004) [61] summarized environmental investigations carried out from 1992 to 2002 on airborne asbestos fibres in Italian schools. Asbestos was found to be present in mainly vinyl floor coverings and in asbestos-cement products; however, in well-maintained buildings, the mean fibre concentration was comparable to concentrations found in outdoor air (0.5 f/L) [61]. As the majority of the asbestos was non-friable, health risks were only considered to be significant when there was damage and/or deterioration of these products, due to repair, renovation and vandalism [61]. The investigators concluded that there was a need for further research on the effects of low or intermediate exposure levels to asbestos and also that there should be regular surveying and monitoring of fibre release with an aim to avoid uncontrolled disturbance of ACM [61].

Environmental exposure pathways can include ACM located in buildings that are not part of the domestic exposure route (i.e., from buildings not occupied by the householder). In Pastuszka’s experimental determination of the emission rate of asbestos fibres from ACM, it was determined that even vibrations or gusts of wind can cause emissions from the elevation of buildings made from asbestos-cement [62]. This study found that mechanical destruction (for example, due to vandalism) had more influence on fibre emission than atmospheric corrosion for externally based ACM in Poland [62]. Fundamentally, the quality of the surface of the ACM exerted the greatest influence on fibre emissions under mechanical impact [62]. The determination of valid and reliable information about the asbestos-related lung cancer and MM risk in the general population exposed in domestic and outdoor (environmental) scenarios has exceeded 25 years of study [63]. Bourgault et al. (2014) [63] assessed the cancer risk for a general population environmentally exposed to asbestos using a dose–response model and environmental measurements from an asbestos mining town in Quebec. The results showed that the lifetime mortality risk (for lung cancer and MM combined) varied between 1.4 and 4.9 per 100,000 persons for an 80-year exposure duration [63]. A more recent literature review and meta-analysis of studies of pleural MM from non-occupational exposure documented an increased risk of 5.4% for household (domestic) and 6.9% for neighbourhood (environmental) exposure pathways across 12 countries [64]. However, Rake et al. (2009) [49] found that there was no overall risk for those living within a mile of a potential environmental hazard (such as an asbestos factory).

**Figure 3:**
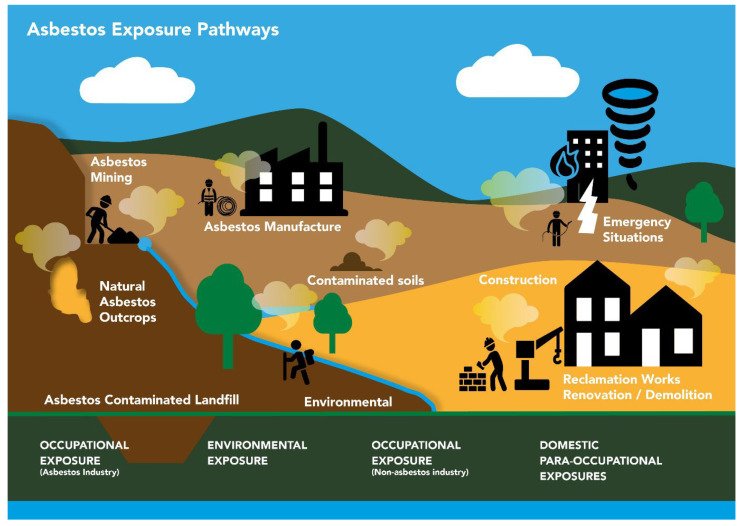
A summary of potential exposure pathways for asbestos exposure globally.

Despite the downturn in the use of asbestos, there are still many opportunities for exposure to occur in an urban environment (Figure 3). Increases in MM cases (to be discussed further in Section 4) demonstrate that the reduction in asbestos mining and manufacturing is linked to alternative pathways, but is there also a danger from other similar EMPs?

### 2.2. Other Elongated Mineral Particles (EMPs)

The number of known exposure pathways for EMPs, such as fibrous erionite has been increasing since 2000 (Table 1), although not all of them have been directly linked to respiratory diseases. Zeolite and zeolitic rocks (e.g., containing erionite) has been mined for various applications, such as for use in ion-exchange processes, road-surfacing, or as adsorbents [26], compositional variability has limited its use in favour of synthetic zeolites and therefore relatively small amounts have been mined in comparison to asbestos. Consequently, potential exposure routes appear to be quite different to those for traditional asbestos minerals.

The use of fibrous erionite-containing materials to cover local roads, parking lots and other areas has received little attention to date, despite evidence that long-term exposure has been linked with MM [67,69]. This has certainly been true for a MM epidemic caused by the EMP of antigorite (belonging to the serpentine mineral group; like chrysotile a phyllosilicate; similar chemical composition, but different crystal structure), that was contained within serpentinite quarry material very commonly used to cover roads [72]. This exposure pathway was also considered where air concentrations of fibrous erionite in cars and school buses transiting on North Dakota roads were found to be equal to or greater than those recorded in the village of Boyali, Turkey, which experienced a 6.5% mortality from MM [17,37]. A direct link to MM has yet to be established for this pathway; however, Wolfe at al. (2017) [73] researched potentially hazardous inhalation risks caused by dust liberated by the use of off-road vehicles in geographic regions where EMPs occur naturally.

In Mexico, lung biopsies from some MM patients have confirmed the presence of fibrous erionite in the samples, which has been attributed to exposure to high levels of zeolitic soils from agricultural activities, such as the tilling of soils, storage of vegetables coated with dust and the use of zeolite-containing materials in agricultural products [67]. In the Lessini mountains of northern Italy, the discovery of fibrous and asbestiform erionite combined with the vast number of quarries and mining activities operating in zeolite host rocks prompted the suggestion that a detailed risk assessment should be carried out, respirable airborne particles should be quantified, and any possible epidemiological evidence should be investigated [45]. A high incidence rate of MM in this region could be a sign of exposure to fibrous and asbestiform erionite, but no detailed study has yet been undertaken. Therefore, exposure to fibrous erionite from earthmoving, agricultural and recreational activities that create dust have to be considered a significant risk in regions with zeolite-rich soils.

In Wyoming, USA, a number of activities specific to landscaping and ground maintenance were analysed for the presence of erionite fibres in air samples [70]. TEM was used with energy dispersive spectroscopy (EDS) and magnification of 20,000× or greater to analyze the erionite samples according to a modified (for erionite) NIOSH Method 7402. Airborne fibrous erionite concentrations ranged from not detected to 0.36 fibres per cubic centimeter (f/cc) erionite but with a reduction in concentration observed during periods of wet deposition (e.g., rain and snow) [70]. Results of the study led the authors to urge the use of personal protective equipment (PPE) and health and safety protocols to prevent dust inhalation or the transmission of dust into vehicles, work or home spaces at sites where erionite fibres may be present. Such a protocol may reduce risks considerably for occupational exposure; however, this may not be the only or predominant exposure pathway.

**Figure 4:**
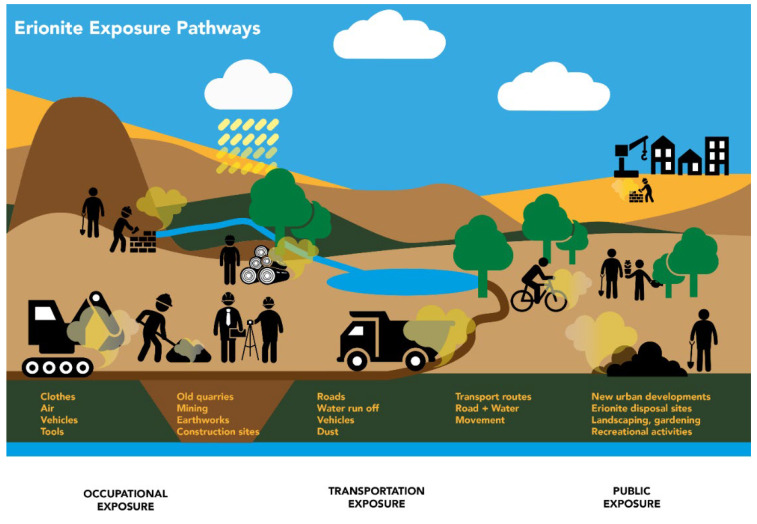
A summary of potential exposure pathways for erionite and other EMPs.

In the past, exposure pathways have been identified as being primarily occupational for asbestos but environmental for fibrous erionite [74]. However, in regions undergoing high levels of community development, e.g., the Auckland Central Business District [75], there may be an occupational risk for engineers and site staff who disturb zeolitic outcrops containing erionite. This is in stark contrast to asbestos minerals, which are now presenting significant environmental exposure pathways. Another exposure pathway that has yet to be investigated is the transferral of excavated materials (containing EMPs) to new regions prior to land development. As demonstrated previously in this article, fibrous erionite has been established in locations worldwide, some of which are relatively remote presently, but our global population is continuing to expand. Figure 4 summarises potential exposure pathways for fibrous erionite exposure which may also be applicable for a number of other EMPs globally.

## 3. Crystallochemistry and Mineralogy of Zeolites

Zeolites are a large group of hydrated aluminosilicates, consisting of about 50 species [76]. Zeolites are characterized by a tectosilicate framework based on SiO_4_ and AlO_4_ tetrahedra, extra-framework cations such as Na^+^, Ca^2+^, K^+^, and variable amounts of H_2_O.

Zeolites may occur mostly with a platy or lamellar habit (characterized by layered crystal structures), fibrous habit (characterized by chain-like crystal structures), or equant habit [77], and the same species may occur with different habits. Notable zeolites that pose or may pose a possible health hazard because they can have fibrous habits are; erionite, ferrierite, mazzite, mesolite, mordenite, natrolite, thomsonite, roggianite, and scolecite [27,76].

Erionite has a general formula of K_2_NaMgCa_1.5_(Al_8_Si_28_)O_72_•28H_2_O, and a structure belonging to the ABC-6 family which is based on stacking along the c-axis of 6-fold ring layer, made up of SiO_4_ and AlO_4_ tetrahedra [74,76]. Erionite is found in diagenetically altered sediments, altered basalt cavities, and hydrothermal alteration areas.

Ferrierite is commonly found in filled vesicles within altered basalts and andesite, and in tuffaceous sediments [78,79]. Ferrierite has a general formula of Na_2_Mg_2_Al_6_Si_30_O_72_•18H_2_O and its framework atoms are commonly arranged in an *Immm* symmetry, based on 5-1 secondary building units [80,81,82].

Mazzite is found in cavities within porphyritic olivine basalts, has a general formula of K_2_CaMg(Si, Al)_36_O_72_•28H_2_O with a framework of SiO_4_ and AlO_4_ tetrahedra having a hexagonal symmetry that can be easily observed in the mineral habit [77,83] and having space group *P6_3_/mmc*.

Mordenite is found as an alteration product of pyroclastic sediment and sedimentary rocks. Mordenite has a general formula of K_2.8_Na_1.5_Ca_2_(Al_9_Si_39_)O_96_•29H_2_O and a framework of SiO_4_ and AlO_4_ tetrahedra arranged in hexagonal sheets [77], with a resulting orthorhombic *Immm* symmetry and 5-1 secondary building units, as for ferrierite [82].

Natrolite can be found in veins and cavities within altered basaltic rocks and as a diagenetic alteration product in sedimentary rocks [84]. Natrolite has a general formula of Na_2_Al_2_Si_3_O_10_•2H_2_O, orthorhombic symmetry *mm2,* and acicular to fibrous morphologies.

Roggianite occurs as a secondary mineral in hydrothermally altered dikes. It has a general formula of Ca_15_(Si, Al, Be)_48_O_90_(OH)_16_•34H_2_O, and its framework has the peculiarity of having BeO_4_ tetrahedra, being the only zeolite with tetrahedrally coordinated Be atoms in its structure [85,86]. The mineral occurs in the habit of thin fibres.

Additional zeolite minerals, not listed here, may pose a health hazard in their growth habit or as a cleavage fragment, and it is thus important to continue monitoring new MM occurrences along with careful characterization of the material of interest. This will be significant as we investigate the trends in global MM cases which continue to rise in many regions of the world and may not be solely due to asbestos.

## 4. Malignant Mesothelioma (MM)

MM is a relatively rare and very severe form of cancer with a highly limited survival rate [87]. Although asbestos can cause a variety of fatal and non-fatal diseases, it is MM, i.e., a cancer of the pleural, peritoneal, pericardial and testicular membranes, that was previously thought to be caused exclusively by exposure to this group of EMPs [88].

A sharp decline in the production of asbestos-containing materials, influenced by the widespread banning of these minerals for industrial applications, may have reduced incidences of long-term exposure in many countries. For example, Canada has dramatically reduced its own consumption of asbestos in recent years, although it continued to export large amounts of the product to countries with less stringent regulations such as India, China and countries in Southeast Asia until 2011 [89], finally banning mining, use, and export of asbestos in 2016 [90]. However, the latency period, the interval between first exposure and the development of ARD, can range from about 25 to 71 years in the case of MM [91]. Such a long latency and a general lack of awareness may be responsible for steady increases in cases of ARDs, including MM (Table 2). In the mid 1990s, the number of MM notifications in Australia were double those of New Zealand MM incidences (for males only) [92], and yet close to 20 years later, cases in Australia have increased by nearly 5-fold [93] compared to an 18-fold increase in New Zealand. In 2004, the WHO estimated that more than 100,000 people died of ARDs [94], and in some countries, exposure to asbestos fibres is the primary cause of occupational death [89]. Currently, about 125 million people in the world are exposed to asbestos in the workplace, and several thousand deaths annually can be attributed to exposure to asbestos in homes [94]. Evidence has also shown that the risk group for ARDs appears to have changed, with fewer incidences attributed to raw fibre handling and more cases attributed to home maintenance and renovation. In addition, non-occupational asbestos exposures contribute an increasing proportion of disease “implicated in up to 30% of cases in the USA and predicted to account for an increasing proportion of the disease” [95]. In some regions, such as Brazil, there is evidence of significant underreporting of mesothelioma cases/deaths by an average of 33% from 2008 to 2014, which may partly explain variations shown in Table 2 [96]. More detailed information about the global burden of mesothelioma per country is provided by Zhai et al. (2021) [97].

Factors affecting the potency of EMPs as carcinogens include particle size, shape, chemistry, high surface area, iron present on the surface of the particle, and in vivo durability (bio-persistence) in the lung tissue [74]. However, determining their potency as human carcinogens is complicated by many factors. For example, there may be subtle differences in the chemistry and morphology in different samples of the same EMP, which are difficult to analyse and there may be a lack of standardised sampling and analytical methods available [70]. Furthermore, EMP population is widely heterogeneous in its chemical composition and dimensional distribution of all the measured dimensional parameters (length in particular has usually larger σ_n-1_ values) [106,107,108].

In vitro studies (performed in cell culture-based assays) demonstrated that erionite was no more cytotoxic than chrysotile and less so than amosite [66]. In contrast, as mentioned earlier, in vivo studies in rats revealed that erionite was much more carcinogenic than asbestos [22,71]. One potential explanation is that while erionite is less cytotoxic than amosite and comparable to chrysotile, it is considerably more mutagenic, pro-proliferative, and cell transforming than asbestos particles, which would explain its high carcinogenicity, as exposed cells are not killed, but instead receive substantial genetic damage that can change the cell behaviour in ways that promote uncontrolled, cancerous growth [66]. This would explain the 500 to 800 times higher carcinogenic potential of erionite-K found in Rome, Oregon samples compared to chrysotile asbestos [30].

Wagner (1982) [71] conducted animal tests using erionite from Oregon, Karain and New Zealand. This study, which introduced erionite to rats via intrapleural injection produced variable results, with 55–60% of the test specimens developing MM using fibrous erionite from Oregon and Karain compared to only 15% from New Zealand fibrous erionite. In addition, 57% of the rats exposed to Oregon fibrous erionite (via inhalation) developed MM compared to 0% using New Zealand fibrous erionite [71]. However, it should be considered that Wagner tested only a single sample of New Zealand fibrous erionite (location not specified), and therefore it is unclear if this lower carcinogenicity is a general characteristic of NZ fibrous erionite perhaps due to a variation in chemical/physical properties and/or associated with the quality of the sample the researchers had available. The use of intrapleural injection (Stanton hypothesis, [109]) has also been questioned as a reliable methodology for these tests [110]. It would be advisable to test samples across a specific geographic region in NZ, including fibrous erionite from weathered and fresh rocks, sediments and exposed rock surfaces which may vary in conditions and carcinogenicity.

The first fibrous erionite-related MM cluster in a community was identified in several villages in Cappadocia, Turkey where the village foundations lay on a zeolite-rich geological unit [111,112]. Cases of pleural and peritoneal MM accounted for 50% of deaths over a 17-year period during the 1970s–1980s [113]. The first confirmed erionite-related cases of MM in North America were identified from two neighbouring states in Mexico, Zacatecas and Jalisco [67]. One question yet unanswered is the effect of acute exposure to high fibrous erionite concentrations in comparison to chronic exposure. Although the exposure in Cappadocia was shown to have directly caused MM, this exposure was thought to be long-term and there was some suggestion of genetic vulnerability amongst the population [68,114].

While hereditary defects, particularly germline *BAP1* mutations, can markedly enhance risk of MM and susceptibility to asbestos carcinogenicity in mice [115,116], to date no germline mutations have been reported in Cappadocian villagers. Moreover, the idea of a genetic component was questioned by Metintas et al. (2010) [69], who characterized the fibres present in mineral samples obtained from stones used in the construction of houses in one of the Cappadocian villages, Karain, and compared them to the occurrence of MM in families there. Their study clearly revealed that all families with high percentages of the family members contracting MM were from houses in a specific part of the village and all these houses were built from Akkusak stone and “Water stone”, which both contain fibrous erionite-filled nodules. All other houses in Karain, including the traditional cave dwellings, were built from and into other rock types, which all tested negative for erionite, and none of the inhabitants of these houses suffered from MM. While this does not rule out a possible contribution by a genetic factor(s), the Metintas et al. (2010) [69] study is strongly supportive of indoor exposure to erionite being the prime explanation for the extreme risk of MM in specific families in Karain. Furthermore, the risk appears to be higher among families exposed to erionite at higher concentrations and/or for longer durations. MM occurred only in people who had lived at least 20 years in fibrous erionite-contaminated houses, including inhabitants who had been born in other villages and married into a Karain family. A plateau for MM risk was reached after 40 years of exposure, likely because other age-related diseases were starting to exert an effect as well [69]. This highlights that fibrous erionite concentration and exposure duration are the major risk factors for erionite-induced MM. Therefore, managing exposure to fibrous erionite seems to be the only proven method to reduce MM risk in erionite-rich regions.

### Future Exposure in Urban Areas

Although the risk of ARDs caused by asbestos mining, use and importation has been reduced by asbestos bans in many countries, risks due to the disturbance of ACM are still significant and ongoing. Community exposure, which results in higher proportions of MM in women and a younger age distribution, may challenge occupational exposure rates [117].

Increased urban development may disturb outcrops of asbestos, fibrous erionite, or soil containing other types of carcinogenic EMPs, leading to more exposure pathways [17,37,72,118,119,120,121]. It is therefore essential to investigate all EMPs, from a variety of geographical locations and under a variety of scenarios to ascertain likely pathways related to ARDs. For example, fibrous erionite has been identified in central urban areas, such as Auckland, New Zealand [75], but has yet to be directly linked to MM cases. The exposure likelihood from fibrous erionite in this region is fundamentally connected to its behaviour as a carcinogen. The direct link to MM in Rome, Oregon, was also not determined; however, in consideration of exposure likelihood, the current population density of Rome, Oregon is 3.64/km^2^ whereas Auckland has a population density of 1210/km^2^. In rapidly developing regions, which have been founded on volcanic substrate, the possibility of a future epidemic due to exposure to EMPs could follow from disturbance of sub-surface clasts of zeolitic material.

In contrast to asbestos, fibrous erionite does not have established occupational exposure limits (OELs) [70]. Despite a study by Jurinski and Jurinski (1997) [122] recommending an 8-h exposure limit of 0.0007 f/cc of air for fibrous erionite over 20 years ago, there remains a lack of standards, sampling methodology, regular airborne fibrous erionite analysis, or regulatory OELs [70,122]. Likewise, there appears to be a general lack of awareness of community and environmental exposure to asbestos [117], despite the more developed legislation compared to other EMPs.

## 5. Conclusions

Human exposure pathways to asbestos have changed considerably, although inconsistently around the world. Although mining activities and the importation of ACM in some countries may have reduced occupational exposure, domestic and environmental exposure pathways are worrying alternatives. The disturbance of asbestos-containing building materials (often via renovation or demolition or reclamation) has been identified as a dominant human risk pathway in many regions, and more recently hazards due to geologically occurring asbestos have been highlighted, alongside other carcinogenic mineral fibres. EMPs have been found to be widely distributed around the world, and there are numerous exposure pathways to humans even when they are not mined for commercial use. Predicting, understanding and being able to identify EMPs that may cause a number of diseases including MM continue to present research challenges. Commercially, asbestos minerals are well-known carcinogens, and global management procedures to deal with asbestos-contaminated buildings have been established. By contrast, environmental exposure or release of dust during earthmoving activities in areas with geologically occurring asbestos or similar EMPs have been less well researched; consequently, there are few management strategies currently instituted. Erionite is a highly carcinogenic EMP that has caused MM outbreaks in Turkey and Mexico. It is found in other regions around the world and has the potential to pose dangers in urban areas with underlying fibrous erionite occurrences.

Auckland, New Zealand has been found to have natural fibrous erionite occurrences that may present a risk when disturbed [75]. Besides natural erosion, increased urban development may disturb outcrops of asbestos-containing rocks, fibrous erionite-rich volcanic rocks, or soil containing these and other types of carcinogenic EMPs, leading to more instances of exposure, and it is, therefore, important that we establish safe protocols for identification, excavation, transportation and disposal of hazardous mineral fibre-contaminated soil. These regulations may be similar to those for asbestos particles from contaminated buildings and land, where the disposal protocol often involves burial within a designated area within a managed refuse disposal site until a more sustainable solution becomes available. It is essential that these strategies are tested specifically for other EMPs before they can be relied on as acceptable processes. Recent research into the bioremediation potential of asbestos-contaminated soil could be relevant for future bioremediation of fibrous erionite and other carcinogenic minerals [54].

Due to the high carcinogenic potential of fibrous erionite compared to asbestos, even low concentrations of fibrous erionite might pose a significant risk. Therefore, all fibrous erionite-containing areas near populated locations and especially cities built on fibrous erionite-containing substrates should be explored to quantify the risk posed by fibrous erionite and where necessary to establish restrictions and procedures to protect both construction workers and the general public from exposure. Safe working environments, transportation protocols and disposal options (which are long-term and sustainable) should be established for volcanic areas globally. Due to the long delay of up to 40 years between fibrous erionite exposure and the onset of MM, a “wait and see” approach could result in fatalities and, therefore, scientifically sound planning should be encouraged in areas that present risks of exposure to erionite. Thus, while in some respects similar to the hazard posed by asbestos in building materials, geologically occurring erionite has specific risk factors that need to be addressed.

## Figures and Tables

**Table 1:** Summary of observed erionite exposure pathways and their link to evidence of MM (form and habit identified where possible).

Location	Erionite Form/Species	Evidence of MM?	Main Exposure Pathways Highlighted
Rome, Oregon, USA	Fibrous, Erionite-K, Erionite-Na, Fe-free [65]	Test organisms (rats) only	Low population density, no exposure described [66]
Zacatecas and Jalisco, Mexico	Not specified	Yes, human fatalities. Erionite confirmed in lung tissue of one MM case [67]	Adobe bricks and terraces [44]; agricultural tilling [67]
Karlik, Cappadocia, Turkey	Fibrous, Erionite-K [68]	Yes, human fatalities; low rate compared to neighbouring Karain [69]	General low concentration; environmental exposure suggested responsible for the relatively low incidence rate of MM in Karlik [69]
Karain, Cappadocia, Turkey	Fibrous, Erionite-K [68]	Yes, human fatalities at extremely high rate [69]	Living in houses built of materials containing erionite nodules [69]
East Gate, Nevada, USA	Not specified	Not specified	Not specified
Dunn County, North Dakota, USA	Not specified	No	Gravel used as building materials, e.g., non-paved roads [65]
Wyoming, South Dakota and Montana, USA	Fibrous, form not specified	No	Campground maintenance, universal terrain vehicle use, tree surgery, digging [70]
Northern Italy, Lessini Mountain area	Fibrous and asbestiform, Erionite-Ca predominantly with Na and K [45]	Elevated MM rates in the general region, but no detailed epidemiological study yet [45]	Mining, quarrying and construction materials [45]
Kandovan, Iran	Fibrous, form not specified	No	Construction and inhabitation of cave dwellings and agricultural use suspected [28]
New Caledonia	Not specified	Not specified	Not specified
New Zealand	Not specified	Test organisms (rats)	Not specified [71]

**Table 2:** Demographics of MM cases worldwide.

Country	No. MM Deaths per Year	Ave. Age at Diagnosis (Years)	Percentage of Population (% × 10^−4^)	No. of MM Cases Cases per Year Timeframe	
New Zealand	100–170 ^^^	50–60 *	23.4–39.8 (2008)	1.8–33	1971–1996
USA	3000	65–74	7.6 (2011)	3200 ^+,^**	2003–2008
Australia	757	70–79	31.0 (2016)	135–631	1982–2017 *
UK	2500	75–79 *	37.4 (2017)	1164–2526	1982–2015 *
Canada	515	60	15.1 (2010)	153–344	1984–2003
China	1659	N/A ^#^	1.2 (2013)	2041	2013
Brazil	142	N/A	0.7 (2010)	N/A	2008–2014
Germany	1480	74–75	18.0 (2016)	1340	2016
Netherlands	481–1000	N/A ^#^	29.0–60.2 (2010)	2587	2008–2012
World	47,000	-	6.7 (2011)	3718–9993 **	1994–2008

Key: ^ uncertainty whether this number includes other asbestos-related cancers; * men only; ** numbers based on mean for timeframe, ^#^ average age of diagnosis not available within data source(s). Note: **^+^** The number of MM cases indicates the increase observed over the timeframe stated, except for the USA, for which there were minimal increases observed over the last several decades. References: [89,92,93,94,98,99,100,101,102,103,104,105].

## References

[1] Lin R.-T., Soeberg M.J., Chien L.-C., Fisher S., Takala J., Lemen R., Driscoll T., Takahashi K. (2018). Bibliometric analysis of gaps in research on asbestos-related diseases: Declining emphasis on public health over 26 years. BMJ Open.

[2] Odgerel C.O., Takahashi K., Sorahan T., Driscoll T., Fitzmaurice C., Yoko-o M., Sawanyawisuth K., Furuya S., Tanaka F., Horie S. (2017). Estimation of the global burden of mesothelioma deaths from incomplete national mortality data. Occup. Environ. Med..

[3] Jablonski R.P., Kim S.J., Cheresh P., Kamp D.W. (2017). Insights into mineral fibre-induced lung epithelial cell toxicity and pulmonary fibrosis. EMU Notes Mineral..

[4] National Institute of Safety and Occupational Health (NIOSH) (2011). Current Intelligence Bulletin 62: Asbestos Fibres and Other Elongate Mineral Particles: State of the Science and Roadmap for Research. Centres for DISEASE Control and Prevention.

[5] Filetti V., Vitale E., Broggi G., Hagnäs M.P., Candido S., Spina A., Lombardo C. (2020). Update of in vitro, in vivo and ex vivo fluoro-edenite effects on malignant mesothelioma: A systematic review. Biomed. Rep..

[6] International Agency for Research on Cancer (IARC) (2012). Arsenic, metals, fibres and dusts. IARC Monographs on the Evaluation of Carcinogenic Risks to Humans.

[7] Bernstein D.M., Rogers R.A., Sepulveda R., Kunzendorf P., Bellmann B., Ernst H., Creutzenberg O., Phillips J.I. (2015). Evaluation of the fate and pathological response in the lung and pleura of brake dust alone and in combination with added chrysotile compared to crocidolite asbestos following short-term inhalation exposure. Toxicol. Appl. Pharmacol..

[8] Linton A., Vardy J., Clarke S., van Zandwijk N. (2014). The ticking timebomb of asbestos: Its insidious role in the development of malignant mesothelioma. Crit. Rev. Oncol. Hematol..

[9] Belluso E., Cavallo A., Halterman D., Gualtieri A.F. (2017). Crystal Habit of Mineral Fibres. Mineral Fibres: Crystal Chemistry, Chemical-Physical Properties, Biological Interaction and Toxicity.

[10] Chatfield E.J. (2018). Measurement of elongate mineral particles: What we should measure and how do we do it?. Toxicol. Appl. Pharmacol..

[11] Veblen D.R., Wylie A.G., Guthrie G.D., Mossman B.T. (1993). Mineralogy of amphiboles and 1:1 layer silicates. Reviews in Mineralogy and Geochemistry.

[12] U.S. Geological Survey (2021). Mineral Commodity Summaries, January 2021. https://pubs.usgs.gov/periodicals/mcs2021/mcs2021-asbestos.pdf.

[13] Frank A.L., Joshi T.K. (2014). The global spread of asbestos. Ann. Glob. Health.

[14] Carlin D.J., Larson T.C., Pfau J.C., Gavett S.H., Shukla A., Miller A., Hines R. (2015). Current research opportunities to address environmental asbestos exposures. Environ. Health Perspect..

[15] Visonà S.D., Capella S., Bodini S., Borrelli P., Villani S., Crespi E., Frontini A., Colosio C., Belluso E. (2021). Inorganic fibre lung burden in subjects with occupational and/or anthropogenic environmental asbestos exposure in Broni (Pavia, Northern Italy): An SEM-EDS study on autoptic samples. Int. J. Environ. Res. Public Health.

[16] Barrett J.C. (1994). Cellular and molecular mechanisms of asbestos carcinogenicity: Implications for biopersistence. Environ. Health Perspect..

[17] Carbone M., Yang H. (2012). Molecular pathways: Targeting mechanisms of asbestos and erionite carcinogenesis in mesothelioma. Clin. Cancer Res..

[18] Gaudino G., Xue J., Yang H. (2020). How asbestos and other fibres cause mesothelioma. Transl. Lung Cancer Res..

[19] Cheresh P., Kim S., Jablonski R.P., Watanabe S., Lu Z., Chi M., Helmin K.A., Gius D., Budinger G.R.S., Kamp D.W. (2021). SIRT3 Overexpression ameliorates asbestos-induced pulmonary fibrosis, mt-DNA damage and lung fibrogenic monocyte recruitment. Int. J. Mol. Sci..

[20] Poole A., Brown R.C., Turver C.J., Skidmore J.W., Griffiths D.M. (1983). In vitro genotoxic activities of fibrous erionite. Br. J. Cancer.

[21] Gualtieri A.F., Gandolfi N.B., Pollastri S., Burghammer M., Tibaldi E., Belpoggi F., Pollok K., Langenhorst F., Vigliaturo R., Dražić G. (2017). New insights into the toxicity of mineral fibres: A combined in situ synchrotron μ-XRD and HR-TEM study of chrysotile, crocidolite, and erionite fibres found in the tissues of Sprague-Dawley rats. Toxicol. Lett..

[22] Wagner J.C., Skidmore J.W., Hill R.J., Griffiths D.M. (1985). Erionite exposure and mesotheliomas in rats. Br. J. Cancer.

[23] Van Gosen B.S., Blitz T.A., Plumlee G.S., Meeker G.P., Pierson P.M. (2013). Geologic occurrences of erionite in the United States: An emerging national public health concern for respiratory disease. Environ. Geochem. Health.

[24] Giacobbe C., Wright J., Dejoie C., Tafforeau P., Berruyer C., Vigliaturo R., Gieré R., Gualtieri A.F. (2019). Depicting the crystal structure of fibrous ferrierite from British Columbia using a combined synchrotron techniques approach. J. Appl. Crystallogr..

[25] Gualtieri A.F., Gandolfi N.B., Passaglia E., Pollastri S., Mattioli M., Giordani M., Ottaviani M.F., Cangiotti M., Bloise A., Barca D. (2018). Is fibrous ferrierite a potential health hazard? Characterization and comparison with fibrous erionite. Am. Mineral..

[26] National Research Council (NRC), Committee on Nonoccupational Health Risks of Asbestiform Fibres, Board on Toxicology and Environmental Health Hazards (1984). Asbestiform Fibres: Nonoccupational Health Risks.

[27] Wright W.W., Rom W.N., Moatamed F. (1983). Characterisation of zeolite fibre sizes using scanning electron microscopy. Arch. Environ. Occup. Health.

[28] Ilgren E.B., Kazemian H., Hoskins J.A. (2015). Kandovan the next ‘Capadoccia’? A potential public health issue for erionite related mesothelioma risk. Epidemiol. Biostatics Public Health.

[29] Ballirano P., Andreozzi G.B., Dogan M., Dogan A.U. (2009). Crystal structure and iron topochemistry of erionite-K from Rome, Oregon, U.S.A. Am. Mineral..

[30] Coffin D.L., Cook P.M., Creason J.P. (1992). Relative mesothelioma induction in rats by mineral fibres: Comparison with residual pulmonary mineral fibre number and epidemiology. Inhal. Toxicol..

[31] Marantos I., Christidis G.E., Ulmanu M., Inglezakis V.J., Zorpas A.A. (2012). Zeolite formation and deposits. Handbook of Natural Zeolites.

[32] Christie A.B., Brathwaite R.L., Thompson B.N. (2002). Mineral Commodity Report 23—Zeolites. N. Z. Min..

[33] Diale P.P., Muzenda E., Zimba J. A study of South African natural zeolites properties and applications. Proceedings of the World Congress on Engineering and Computer Science 2011, Vol II, WCECS 2011.

[34] Batiashvili T.V., Gvakharia G.V. (1968). Erionite found for the first time in Georgia. Dokl. Russ. Acad. Sci. Earth Sci. Sect..

[35] Lehtinen M. (1976). Lake Lappajärvi, a Meteorite Impact Site in Western Finland. Geological Survey of Finland Bulletin.

[36] Tschernick R.W. (1992). Zeolites of the World.

[37] Carbone M., Baris Y.I., Bertino P., Brass B., Comertay S., Dogan A.U., Gaudino G., Jube S., Kanodia S., Partridge C.R. (2011). Erionite exposure in North Dakota and Turkish villages with mesothelioma. Proc. Natl. Acad. Sci. USA.

[38] Galli E., Quartieri S., Vezzalini G., Alberti A. (1996). Gottardiite, a new high-silica zeolite from Antarctica: The natural counterpart of synthetic NU-87. Eur. J. Minerol..

[39] Surdam R.C., Eugster H.P. (1976). Mineral reactions in the sedimentary deposits of the Lake Magadi region, Kenya. Geol. Soc. Am. Bull..

[40] Noh J.H., Kim S.J. (1986). Zeolites from tertiary tuffaceous rocks in Yeongil area, Korea. Stud. Surf. Sci. Catal..

[41] Passaglia E., Artioli G., Gualtieri A. (1998). Crystal chemistry of the zeolites erionite and offretite. Am. Mineral..

[42] Suprychev V.A., Prokhorov I.G. (1986). Erionite from keratophyre volkanites of the Karadag Reserve in the Crimea. Mineral. Sb..

[43] Kirov G., Samajova E., Nedialkov R., Stanimirova T.S. (2011). Alteration processes and products of acid pyroclastic rocks in Bulgaria and Slovakia. Clay Miner..

[44] Saracci R. (2015). Erionite and cancer in a Mexican village. Occup. Environ. Med..

[45] Giordani M., Mattioli M., Ballirano P., Pacella A., Cenni M., Boscardin M., Valentini L. (2017). Geological occurrence, mineralogical characterisation, and risk assessment of potentially carcinogenic erionite in Italy. Toxicol. Environ. Health Part B Crit. Rev..

[46] Mattioli M., Giordani M., Arcangeli P., Valentini L., Boscardin M., Pacella A., Ballirano P. (2018). Prismatic to asbestiform offretite from Northern Italy: Occurrence, morphology and crystal-chemistry of a new potentially hazardous zeolite. Minerals.

[47] Sahmel J., Barlow C.A., Simmons B., Gaffney S.H., Avens H.J., Madl A.K., Henshaw J., Lee R.J., Van Orden D., Sanchez M. (2014). Evaluation of Take-Home Exposure and Risk Associated with the Handling of Clothing Contaminated with Chrysotile Asbestos. Risk Anal..

[48] Kazan-Allen L. (2019). Chronology of National Asbestos Bans. http://www.ibasecretariat.org/chron_ban_list.php.

[49] Rake C., Gilham C., Hatch J., Darnton A., Hodgson J., Peto J. (2009). Occupational, domestic and environmental mesothelioma risks in the British population: A case-control study. Br. J. Cancer.

[50] Scarselli A., Marinaccio A., Corfiati M., Di Marzio D., Iavicoli S. (2020). Occupational asbestos exposure after the ban: A job exposure matrix developed in Italy. Eur. J. Public Health.

[51] Bard D., Burdett G. (2007). Exposure of UK Industrial Plumbers to Asbestos, Part II: Awareness and Responses of Plumbers to Working with Asbestos During a Survey in Parallel with Personal Sampling. Ann. Occup. Hyg..

[52] Singh R., Cherrie J.W., Rao B., Asolekar S.R. (2020). Assessment of the future mesothelioma disease burden from past exposure to asbestos in ship recycling yards in India. Int. J. Hyg. Environ. Health.

[53] Lemen R.A., Landrigan P.J. (2021). Sailors and the Risk of Asbestos-Related Cancer. Int. J. Environ. Res. Public Health.

[54] Wallis S.L., Emmett E.A., Hardy R., Casper B.B., Blanchon D.J., Testa J.R., Menges C.W., Gonneau C., Jerolmack D.J., Seiphoori A. (2020). Challenging global waste management—bioremediation to detoxify asbestos. Front. Environ. Sci..

[55] Landrigan P.J. (1991). The third wave of asbestos disease: Exposure to asbestos in place—Public health control. Introduction. Ann. N. Y. Acad. Sci..

[56] Olsen N.J., Franklin P.J., Reid A., De Klerk N.H., Threlfall T.J., Shilkin K., Musk B. (2011). Increasing incidence of malignant mesothelioma after exposure to asbestos during home maintenance and renovation. Med. J. Aust..

[57] WorkSafe (2016). Approved Code of Practice: Management and Removal of Asbestos. https://www.worksafe.govt.nz/topic-and-industry/asbestos/management-and-removal-of-asbestos/.

[58] National Occupational Health and Safety Commission (NOHSC) (2005). Code of Practice for the Management and Control of Asbestos in Workplaces. https://www.safetyusb.online/documents/Asbestos/SAFEWORKAUSTRALIA-COP-MgtControlofAsbestosInTheWorkplace_NOHSC2018-2005_PDF.pdf.

[59] Lee R.J., Van Orden D.R. (2008). Airborne asbestos in buildings. Regul. Toxicol. Pharmacol..

[60] Lee E.S., Kim Y.K. (2021). Asbestos Exposure Level and the Carcinogenic Risk Due to Corrugated Asbestos-Cement Slate Roofs in Korea. Int. J. Environ. Res. Public Health.

[61] Campopiano A., Casciardi S., Fioravanti F., Ramires D. (2004). Airborne asbestos levels in school buildings in Italy. J. Occup. Environ. Hyg..

[62] Pastuszka J.S. (2009). Emission of airborne fibers from mechanically impacted asbestos-cement sheets and concentration of fibrous aerosol in the home environment in Upper Silesia, Poland. J. Hazard. Mater..

[63] Bourgault M.H., Gagne M., Valcke M. (2014). Lung cancer and mesothelioma risk assessment for a population environmentally exposed to asbestos. Int. J. Hyg. Environ. Health.

[64] Marsh G.M., Riordan A.S., Keeton K.A., Benson S.M. (2017). Non-occupational exposure to asbestos and risk of pleural mesothelioma: Review and meta-analysis. Occup. Environ. Med..

[65] Ballirano P., Cametti G. (2015). Crystal chemical and structural modifications of erionite fibres leached with simulated lung fluids. Am. Mineral..

[66] Bertino P., Marconi A., Palumbo L., Bruni M., Barbone D., Germano S., Dogan A.U., Tassis G.F., Porta C., Mutti L. (2007). Erionite and asbestos differently cause transformation of human mesothelial cells. Int. J. Cancer.

[67] Ilgren E.B., Pooley F.D., Larragoitiac J.C., Talamantes M., Navarretee G.L., Krauss E., Brena A.F. (2008). First confirmed erionite related mesothelioma in North America. Indoor Built Environ..

[68] Dogan A.U., Baris Y.I., Dogan M., Emri S., Steele I., Elmishad A.G., Carbone M. (2006). Genetic predisposition to fibre carcinogenesis causes a mesothelioma epidemic in Turkey. Cancer Res..

[69] Metintas M., Hillerdal G., Metintas S., Dumortier P. (2010). Endemic malignant mesothelioma: Exposure to erionite is more important than genetic factors. Arch. Environ. Occup. Health.

[70] Beaucham C., King B., Feldmann K., Harper M., Dozier A. (2018). Assessing occupational erionite and respirable crystalline silica exposure among outdoor workers in Wyoming, South Dakota, and Montana. J. Occup. Environ. Hyg..

[71] Wagner J.C. (1982). Health hazards of substitutes. Asbestos, Health & Safety, Proceedings of the World Symposium on Asbestos, Montreal, QC, Canada, 25–27 May 1982.

[72] Baumann F., Maurizot P., Mangeas M., Ambrosi J.P., Douwes J., Robineau B. (2011). Pleural mesothelioma in New Caledonia: Associations with environmental risk factors. Environ. Health Perspect..

[73] Wolfe C., Buck B., Miller A., Lockey J., Weis C., Weissman D., Jonesi A., Ryan P. (2017). Exposure to naturally occurring mineral fibres due to off-road vehicle use: A review. Int. J. Hyg. Environ. Health.

[74] Matassa R., Familiari G., Relucenti M., Battaglione E., Downing C., Pacella A., Cametti G., Ballirano P. (2015). A deep look into erionite fibres: An electron microscopy investigation of their self-assembly. Sci. Rep..

[75] Brook M.S., Black P.M., Salmond J., Dirks K.N., Berry T.-A., Steinhorn G. (2020). Erionite in Auckland bedrock and malignant mesothelioma: An emerging public and occupational health hazard?. N. Z. Med. J..

[76] Gottardi G., Galli E. (1985). General Information on Zeolites. Natural Zeolites.

[77] Klein C., Guthrie G.D., Mossman B.T. (1993). Rocks, minerals and a dusty world. Health Effects of Mineral Dusts.

[78] Regis A.J. (1970). Occurrences of ferrierite in altered pyroclastics in central Nevada. Geol. Soc. Am. Abstr. Programs.

[79] Wise W.S., Tschernick R.W. (1976). Chemical composition of ferrierite. Am. Mineral..

[80] Vaughan P.A. (1966). The crystal structure of the zeolite ferrierite. Acta Crystallogr..

[81] Meier W.M. (1968). Zeolite structures. S.C.I. Monograph, Molecular Sieves.

[82] Smith B.K. (1986). Variations in the framework structure of the zeolite ferrierite. Am. Mineral..

[83] Galli E., Passaglia E., Pongiluppi D., Rinaldi R. (1974). Mazzite, a new mineral, the natural counterpart of the synthetic zeolite. Contrib. Mineral. Petrol..

[84] Deer A., Howie R., Wise W.S., Zussman J. (2004). Rock Forming Minerals. Rock Forming Minerals. Volume 4B. Framework Silicates: Silica Minerals. Feldspathoids and the Zeolites.

[85] Galli E. The crystal structure of roggianite, a zeolite-like silicate. Proceedings of the 5th International Conference of Zeolites.

[86] Passaglia E., Vezzalini G. (1988). Roggianite: Revised chemical formula and zeolitic properties. Mineral. Mag..

[87] Amin W., Linkov F., Landsittel D.P., Silverstein J.C., Bshara W., Gaudioso C., Feldman M.D., Pass H.I., Melamed J., Friedberg J.S. (2018). Factors influencing malignant mesothelioma survival: A retrospective review of the National Mesothelioma Virtual Bank cohort. F1000 Res..

[88] Carbone M., Ly B.H., Dodson R.F., Pagano I., Morris P.T., Dogan U.A., Gazdar A.F., Pass H., Yang H. (2011). Malignant mesothelioma: Facts, myths and hypotheses. J. Cell. Physiol..

[89] Povtak T. (2012). Canada Closing Its Chrysotile Institute, Signaling End of Country’s Asbestos Industry. https://www.asbestos.com/news/2012/04/30/canada-chrysotile-institute-asbestos/.

[90] Ruff K. (2017). How Canada changed from exporting asbestos to banning asbestos: The challenges that had to be overcome. Int. J. Environ. Res. Public Health.

[91] Bianchi C., Bianchi T., Tommasi M. (2007). Mesothelioma of the pleura in the Province of Trieste. Med. Lav..

[92] Kjellstrom T. (2000). Increased mesothelioma incidence in New Zealand: The asbestos-cancer epidemic has started. N. Z. Med. J..

[93] Australian Institute of Health & Welfare (AIHW) (2019). Mesothelioma in Australia 2018. Safe Work Australia, Australian Government. https://www.aihw.gov.au/getmedia/7df8ff10-d0b7-4d42-881b-76647a9263ef/aihw-can-130-infocus_1.pdf.aspx?inline=true.

[94] World Health Organization (WHO) (2020). Chemical Safety and Health—Asbestos. https://www.who.int/teams/environment-climate-change-and-health/chemical-safety-and-health/health-impacts/chemicals/asbestos.

[95] Robinson B.M. (2012). Malignant pleural mesothelioma: An epidemiological perspective. Ann. Cardiothorac. Surg..

[96] Santana V.S., Salvi L., Cavalcante F., Campos F., Algranti E. (2021). Underreporting of mesothelioma, asbestosis and pleural plaques in Brazil. Occup. Med..

[97] Zhai Z., Ruan J., Zheng Y., Xiang D., Li N., Hu J., Shen J., Deng Y., Yao J., Zhao P. (2021). Assessment of Global Trends in the Diagnosis of Mesothelioma From 1990 to 2017. JAMA Netw. Open.

[98] Delgermaa V., Takahashi K., Park E.-K., Le G.V., Hara T., Sorathan T. (2011). Global mesothelioma deaths reported to the World Health Organization between 1994 and 2008. Bull. World Health Organ..

[99] Franz F. (2013). Study Revisits Health Risk of Chrysotile: Why Is This Still a Debate in 2013?. https://www.asbestos.com/news/2013/02/01/health-risk-of-chrysotile/.

[100] Health & Safety Executive (HSE) (2019). Mesothelioma Statistics for Great Britain, 2019. https://www.hse.gov.uk/Statistics/causdis/mesothelioma/mesothelioma.pdf..

[101] Selby K. (2021). Mesothelioma in Canada. https://www.asbestos.com/mesothelioma/canada/.

[102] Bray F., Colombet M., Mery L., Piñeros M., Znaor A., Zanetti R., Ferlay J. (2021). Cancer Incidence in Five Continents, Vol. XI.

[103] Zhao J., Zuo T., Zheng R., Zhang S., Zeng H., Xia C., Yang Z., Chen W. (2017). Epidemiology and trend analysis on malignant mesothelioma in China. Chin. J. Cancer.

[104] Schonfeld S.J., McCormack V., Rutherford M.J., Schüz J. (2014). Regional variations in German mesothelioma mortality rates: 2000–2010. Cancer Causes Control.

[105] Robert Koch Institut (2020). Cancer in Germany 2015/2016. https://www.krebsdaten.de/Krebs/EN/Content/Publications/Cancer_in_Germany/cancer_chapters_2015_2016/cancer_germany_2015_2016.pdf?__blob=publicationFile.

[106] Vigliaturo R., Ventura G.D., Choi J.K., Marengo A., Lucci F., O’Shea M.J., Perez-Rodriguez I., Giere R. (2018). Mineralogical characterization and dissolution experiments in Gamble’s solution of tremolitic amphibole from Passo di Caldenno (Sondrio, Italy). Minerals.

[107] Vigliaturo R., Choi J.K., Pérez-Rodriguez I., Gieré R. (2020). Dimensional distribution control of elongate mineral particles for their use in biological assays. MethodsX.

[108] Vigliaturo R., Elkassas S.M., Ventura G.D., Redhammer G.J., Ruiz-Zepeda F., O’Shea M.J., Drazic G., Giere R. (2021). Multi-scale characterization of glaucophane from Chiavolino (Biella, Italy): Implications for international regulations on elongate mineral particles. Eur. J. Mineral..

[109] Stanton M.F., Layard M., Tegeris A., Miller E., May M., Morgan E., Smith A. (1981). Relation of particle dimension to carcinogenicity in amphibole asbestoses and other fibrous minerals. J. Natl. Cancer Inst..

[110] Nolan R.P., Langer A.M. (1993). Limitations of the Stanton hypothesis. Rev. Mineral..

[111] Baris Y.I., Sahin A.A., Ozesmi M., Kerse I., Ozen E., Kolacan B., Altinörs M., Göktepeli A. (1978). An outbreak of pleural mesothelioma and chronic fibrosing pleurisy in the village of Karain/Urgüp in Anatolia. Thorax.

[112] Baris Y.I., Saracci R., Simonato L., Skidmore J.W., Artvinli M. (1981). Malignant mesothelioma and radiological chest abnormalities in two villages in Central Turkey. An epidemiological and environmental investigation. Lancet.

[113] Niklinski J., Niklinska W., Chyczewska E., Laudanski J., Naumnik W., Chyczewski L., Pluygers E. (2004). The epidemiology of asbestos-related diseases. Lung Cancer.

[114] Roushdy-Hammady I., Siegel J., Emri S., Testa J.R., Carbone M. (2001). Genetic-susceptibility factor and malignant mesothelioma in the Cappadocian region of Turkey. Lancet.

[115] Testa J.R., Cheung M., Pei J., Below J.E., Tan Y., Sementino E., Cox N.J., Dogan A.U., Pass H.I., Trusa S. (2011). Germline BAP1 mutations predispose to malignant mesothelioma. Nat. Genet..

[116] Xu J., Kadariya Y., Cheung M., Pei J., Talarchek J., Sementino E., Tan Y., Menges C.W., Cai K.Q., Litwin S. (2014). Germline mutation of Bap1 accelerates development of asbestos-induced malignant mesothelioma. Cancer Res..

[117] Emmett E.A. (2021). Asbestos in high-risk communities: Public health implications. Int. J. Environ. Res. Public Health.

[118] Carbone M., Emri S., Dogan A.U., Steele I., Tuncer M., Pass H.I., Baris Y.I. (2007). A mesothelioma epidemic in Cappadocia: Scientific developments and unexpected social outcomes. Nat. Rev. Cancer.

[119] Paoletti L., Batisti D., Bruno C., Di Paola M., Gianfagna A., Mastrantonio M., Nesti M., Comba P. (2000). Unusually high incidence of malignant pleural mesothelioma in a town in eastern Sicily: An epidemiological and environmental study. Arch. Environ. Occup. Health.

[120] Pan X.L., Day H.W., Wang W., Beckett L.A., Schenker M.B. (2005). Residential proximity to naturally occurring asbestos and mesothelioma risk in California. Am. J. Respir. Crit. Care Med..

[121] Maher B. (2010). Epidemiology: Fear in the dust. Nature.

[122] Jurinski J.B., Jurinski N.B. (1997). A proposed control limit for exposure to airborne erionite fibres. Appl. Occup. Environ. Hyg..

